# Inflammatory cap polyp of the sigmoid colon: a case report

**DOI:** 10.1186/s13256-021-02857-8

**Published:** 2021-05-29

**Authors:** Narendra Pandit, Tek Narayan Yadav, Mona Dahal, Laligen Awale, Shailesh Adhikary

**Affiliations:** 1 Division of Surgical Gastroenterology, Department of Surgery, B. P. Koirala Institute of Health Sciences (BPKIHS), Dharan, Nepal; 2 Department of Pathology, B. P. Koirala Institute of Health Sciences (BPKIHS), Dharan, Nepal

**Keywords:** Inflammatory polyp, Colon, Intussusceptions, Fibrinopurulent exudates

## Abstract

**Background:**

Inflammatory cap polyp is a very rare benign entity of the distal left colon, characterized by inflammatory polyp with a “cap” of fibrinopurulent exudates. They are usually multiple and commonly present with bleeding per rectum or mucoid discharge. Solitary polyp presenting with intermittent intussusceptions is rare.

**Case presentation:**

We report the case of a 45-year-old Nepalese male with a solitary inflammatory sigmoid colon polyp. The patient presented with a 1-month history of rectal bleeding, mucoid discharge, and severe colicky abdominal pain due to intussusceptions. On colonoscopy, there was an exophytic mass with surface exudates. Colonic resection and anastomosis were performed, due to recurring partial intestinal obstruction. At a 6-month follow-up, the patient was asymptomatic.

**Conclusion:**

Inflammatory cap polyp is a benign entity, and it should be kept in mind as an important differential diagnosis of exophytic colonic mass with surface exudates.

## Introduction

Inflammatory cap polyp is a rare, benign entity of the colon characterized by an inflammatory polyp with a “cap” of fibrinopurulent exudates [1]. The disease was first described by Sir Williams *et al.* [2] in 1985. Since then, fewer than 100 cases have been described in the English literature. Its etiopathogenesis and the best treatment modality are poorly described because of the unfamiliarity and its rarity. They are usually multiple but can be solitary and confined to the left distal colon [3, 4]. Here, we describe an interesting case of a solitary inflammatory cap polyp, which preoperatively mimicked malignancy and required colonic resection for intussusceptions caused by the lesion.

## Case report

**Fig. 1:**
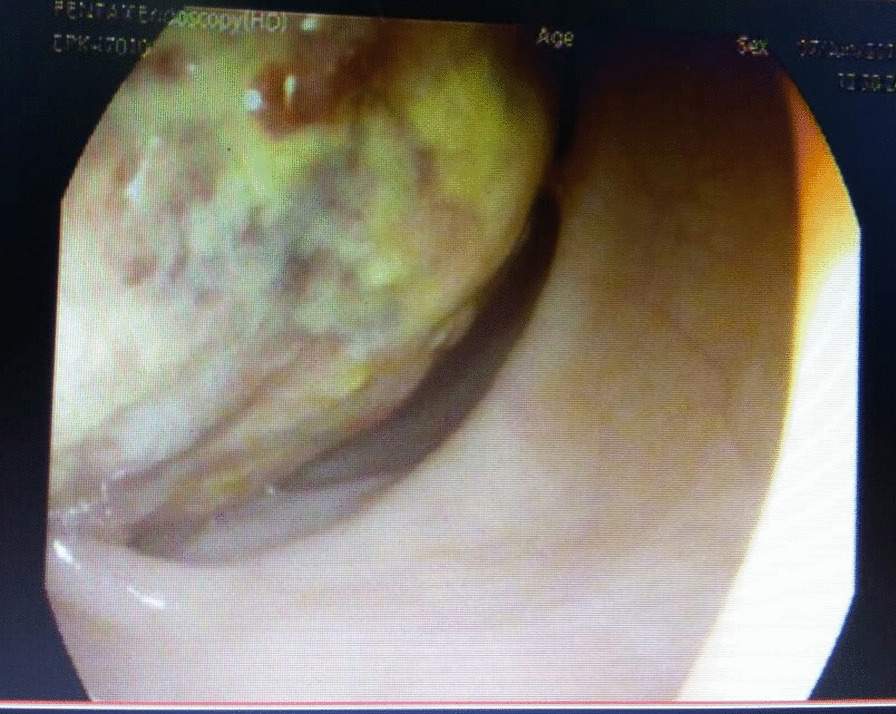
Colonoscopy showing exophytic mass with surface exudates at the left distal colon

**Fig. 2:**
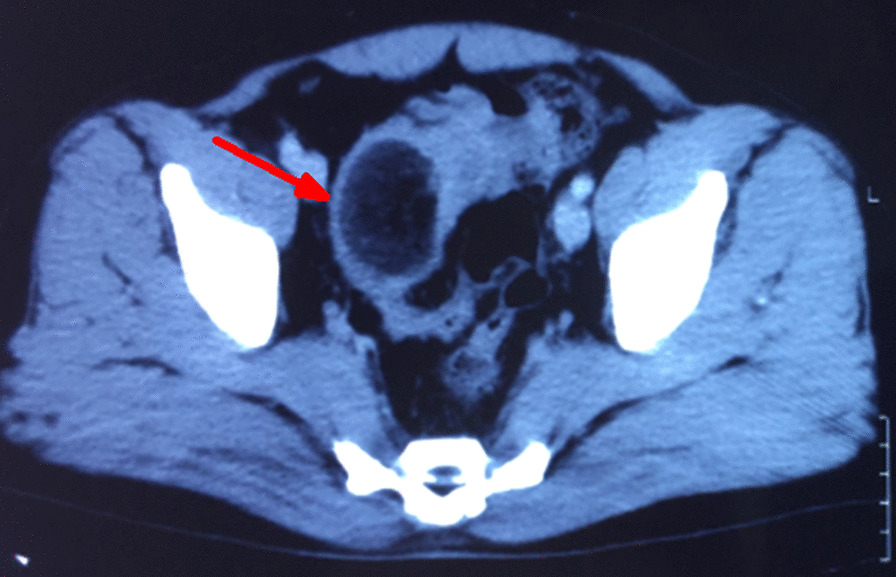
CT abdomen showing well-defined hypodense mass lesion with fat density in the wall of the sigmoid colon causing intussusceptions (*arrow*)

A 45-year-old Nepalese male presented with abdominal pain and blood-mixed stool for 1 month. The pain was located in the left iliac fossa, intermittent, colicky, and at times requiring injectable analgesics for relief. He also complained of the passage of large amounts of mucopurulent discharge, per rectum. He denied any past medical illness and loss of weight or appetite. Moreover, there was no history of smoking or alcohol consumption or family history of inflammatory bowel disease or any gastrointestinal illness. General physical examination was normal. Abdominal and digital rectal examination was also normal, with no palpable lump or tenderness. Blood investigations revealed normal hemoglobin (11.8 gm/dl) and biochemistry. Colonoscopy showed an exophytic mass (size 5 × 5 cm) at descending/sigmoid colon junction that occupied half of the lumen with surface exudates and erosions (Fig. 1). The scope could not be determined further. Tumor marker carcinoembryonic antigen (CEA) was within a normal range (1.69 ng/ml). The patient underwent computed tomography (CT) of the abdomen, which revealed a well-defined hypodense lesion (size 4.6 × 3.4 cm), with a fat density in the wall of the sigmoid colon protruding into the lumen. There was evidence of invagination of descending colon into the sigmoid colon forming a concentric mass suggesting intussusceptions (Fig. 2).

**Fig. 3:**
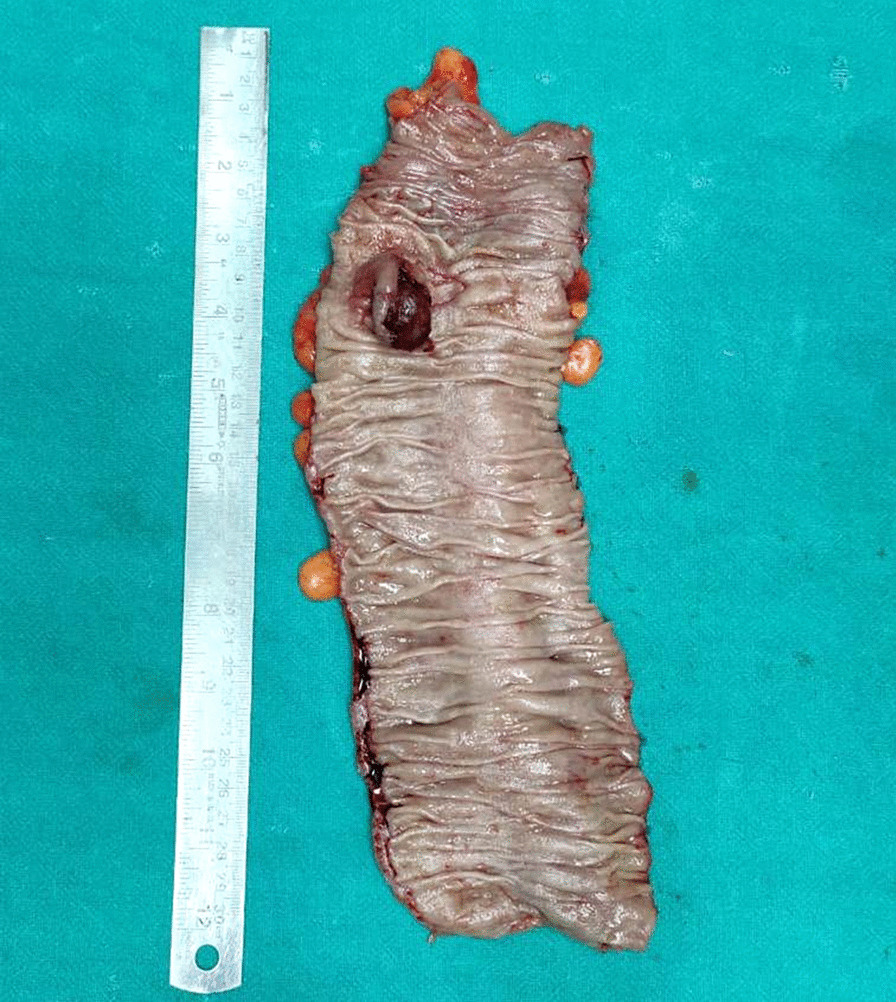
Resected colon showing a reddish, soft polyp with normal intervening colonic mucosa

**Fig. 4:**
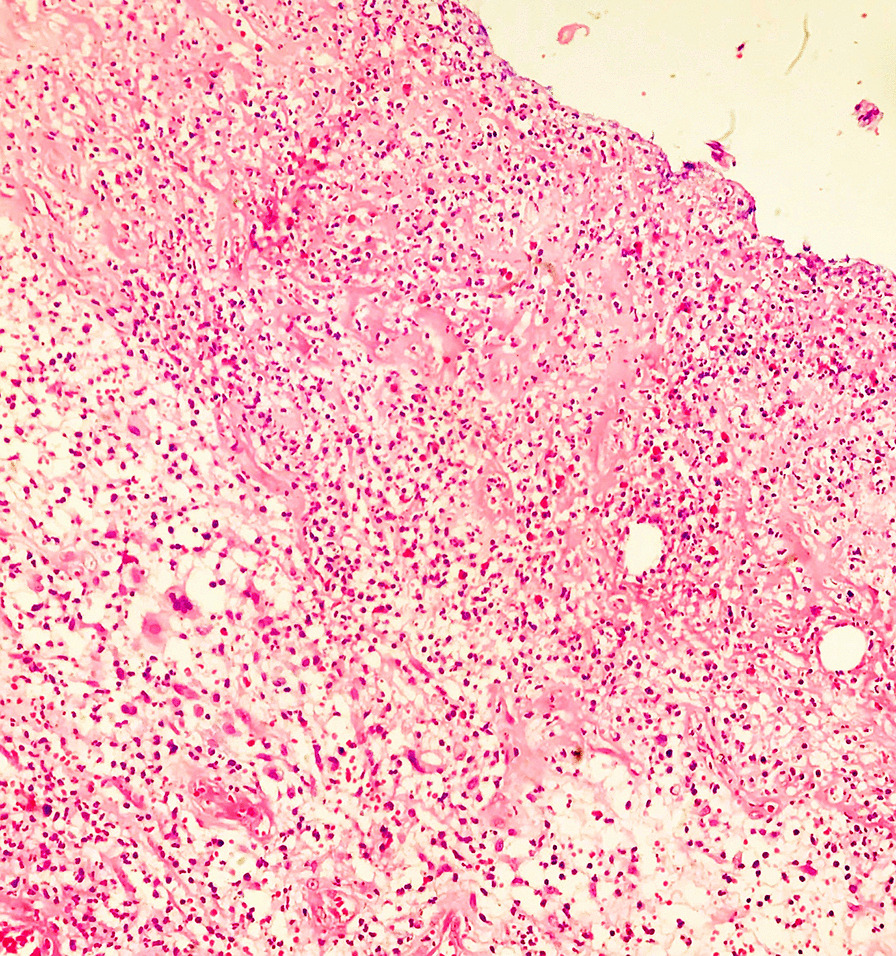
Photomicrograph (*H&E;* × *200*) showing inflammatory cell infiltrates predominantly of neutrophils in the submucosal layer, with a “cap” of fibrinous exudates. No atypical cells seen

Since the time of colonoscopy, the patient had already been placed on a liquid diet, as part of bowel preparation. The decision to opt for an operative procedure, without obtaining a histopathological diagnosis, was made in view of the fact that the patient had recurring pain, suggesting intussusceptions with a lead point of the tumor. At surgery, there were no features of metastases. There was an intraluminal tumor at the sigmoid colon, which was resected with a wide margin and was anastomosed in two layers. Cut-section of the resected colon revealed a reddish, soft polypoidal lesion (Fig. 3). The postoperative period was uneventful, and the patient was discharged on day 5. Histopathological examination of the lesion showed polypoid tissue revealing surface ulceration, capped by fibrinous exudates. The submucosa contained adipocytes and mixed inflammatory cells infiltrate comprising neutrophils, lymphocytes, plasma cells, and eosinophils. The adjacent intestinal wall also showed inflammatory cell infiltrates with an area of cryptitis. The histological features were consistent with the diagnosis of inflammatory cap polyp (Fig. 4). Six months later, the patient continues to remains asymptomatic.

## Discussion

Cap polyp is a rare, nonneoplastic condition that primarily involves the distal colon and the rectum, characterized by the presence of inflammatory polyps with a cap of granulation tissue [5]. They are usually multiple (90%) but can present as a solitary sessile polyp as seen in our patient [6]. The disease is a new and still undefined entity among gastroenterologists and surgeons. It usually occurs in the fifth decade of life, but can often occur at the extremes of ages (1–76 years) [2, 4].

The pathogenesis of cap polyp is controversial, but it has been hypothesized to be caused by dysbiosis of gut microbiota, chronic mucosal irritation caused by straining at defecation, *Helicobacter pylori* infection, mucosal prolapse secondary to colonic dysmotility, and previous pelvic surgery [6, 7]. The most common symptoms at presentation are rectal bleeding (82%), mucoid diarrhea (46%), constipation (64%), and abdominal pain (40%) [3, 5]. Sometimes, the patient can present with intermittent intestinal obstruction due to the intussusceptions caused by a large sessile polyp, as seen in the present case. Diagnosis is established by colonoscopy that characteristically shows a “cap” of fibrinopurulent exudates on it. The entity is further confirmed by biopsy [8]. Biopsy typically shows polyp with elongated, distended, tortuous, and hyperplastic crypts that become attenuated at the mucosal surface. The polyp contains a large number of inflammatory cells in the lamina propria. Most importantly, the surface (“cap”) of the polyp is ulcerated and covered with fibrinopurulent exudates [1, 6].

The main differential diagnosis is pseudopolyp of pseudomembranous colitis (PMC), inflammatory bowel disease (which has a normal colonic mucosa), and the adenomatous/malignant polyps covered by the exudates [2, 9]. In PMC, focal pseudomembranes can coalesce to involve large areas of mucosa to form polyp as the disease progresses. Moreover, it is very important to exclude malignancy, which is a much more common entity. Furthermore, fecal calprotectin and stool *H. pylori* antigen status should also be evaluated to exclude inflammatory bowel disease and infectious origin polyps [6, 7, 9].

The optimal treatment of cap polyp/polyposis is not known; however, a trial of medical therapy should be given to all patients initially [4]. This includes colonoscopy and polypectomy (snare or argon plasma coagulation) [3, 4]. Complete polypectomy should be done whenever possible. It should be followed by the use of laxatives and biofeedback therapy. Surgery with colonic resection should be considered in patients presenting with severe symptoms (obstruction or bleeding), failure of medical treatment, recurrent disease after colonoscopic polypectomy, or inability to exclude malignancy [3, 6]. Other treatment modalities that have been described include watchful waiting in minimally symptomatic patients, metronidazole, steroids, *H. pylori* eradication therapy, and infliximab [2, 10].

Recurrence of the disease is possible and has been described. Hence, postoperative close clinical follow-up or surveillance after colonoscopic polypectomy is necessary to exclude recurrence.

## Conclusion

Inflammatory cap polyp is a rare, benign polyp of the left side of the colon and the rectum. It can present with intussusceptions and, hence, should be kept in mind by the treating physicians when they encounter a “cap” of fibrinopurulent exudates on the polyp on colonoscopy.

## Data Availability

All data generated or analyzed during this study are included in this published article.
